# A Physical Health Profile of Youths Living with a “Hikikomori” Lifestyle

**DOI:** 10.3390/ijerph15020315

**Published:** 2018-02-11

**Authors:** John W. M. Yuen, Yoyo K. Y. Yan, Victor C. W. Wong, Wilson W. S. Tam, Ka-Wing So, Wai Tong Chien

**Affiliations:** 1 School of Nursing, The Hong Kong Polytechnic University, Hung Hom, Kowloon, Hong Kong, China; yoyo.ky.yan@polyu.edu.hk (Y.K.Y.Y.); wai.tong.chien@polyu.edu.hk (W.-T.C.); 2 Department of Social Work, Hong Kong Baptist University, Kowloon Tong, Kowloon, Hong Kong, China; vicwong@hkbu.edu.hk; 3 Alice Lee Centre for Nursing Studies, National University of Singapore, Lower Kent Ridge Road, Singapore 119077, Singapore; wilson_tam@nuhs.edu.sg; 4 Withdrawal Youth Service, Hong Kong Christian Service, Tsim Sha Tsui, Kowloon, Hong Kong, China; sokawing@hkcs.org

**Keywords:** hikikomori, hidden youth, social withdrawal, health, hypertension, obesity, adolescent, physical health

## Abstract

A cross-sectional study was designed to understand the impacts of “hikikomori” lifestyle on physical health. A total of 104 eligible hikikomori cases were recruited from the social services network of Hong Kong with a mean age of 19.02 ± 3.62 (ranged 13–31) year-old, and had completed the set of questionnaires and a series of anthropometric and physical health measurements. Despite SF36 score of 84.0 indicated good physical functioning in general, participants were lived sedentarily with high incidence of hypertension at 15.4% and prehypertension at 31.7%. Occurrence of hypertension and prehypertension in cases living as hikikomori >6 months were 3 times and 1.5 times higher than those newly onset cases, respectively. The blood pressure levels were correlated with age and all obesity index parameters measured including waist circumference and body mass index. Results also observed a shift of body weight from underweight to overweight and obesity along the hikikomori duration. Half of the hypertensive cases involved the elevation of systolic blood pressure, which suggested higher odds of cardiovascular complications. In conclusion, the hikikomori lifestyle could be a risk behavior that may harm the younger generation physically by promoting obesity and hypertension and probably other chronic illnesses.

## 1. Introduction

“Hikikomori” represents a severe form of social withdrawal, typically involving hidden youths experiencing protracted periods of hermitic life at home. The “hikikomori” pandemic has alerted public health experts worldwide, particularly regarding the psychological well-being of these younger generations [1,2]. In Japan, the lifetime prevalence rate of hikikomori was estimated as 1.2% [3], which was comparable with the prevalence of 1.9% reported in Hong Kong according to a recent telephone-based survey [4]. Such local prevalence was close to the initial estimation made by a non-governmental organization that projected 18,500 hikikomori cases (accounted for 2.1% of its youth population) were living in the city [5]. The universal definition includes any individual who without a clear or legitimate purpose, confines themselves at home for more than 6 months, avoiding face-to-face contact with others except family or a close person, and having a ‘Status Zero’—meaning not participating in any in education, training, or work activities [6]. Hikikomori cases have been identified in numerous Western [7,8,9] and Asian countries [3,6,10]. Most cases are discovered during their early-20s, while the earliest onset could be during the junior high school period [5,11,12,13]. Although the etiology remains largely unknown, many researchers believed that this is a personalized and culturally driven phenomenon [14,15]. A few studies [10,16,17,18] had reported low self-esteem characteristics of hikikomori and many of these cases were living unhappily with multiple psychiatric co-morbidities. Particularly in Japan, the incidence of mental disorders occurring in hikikomori was almost twice that of the age-matched population, and the risk of mood disorders was six-times higher among the hikikomori [3]. There is nothing fundamentally wrong with individuals who choose to withdraw from a life that they feel is stressful, and for some a hikikomori lifestyle may be an ideal one, but healthcare professionals are concerned about the kind of lifestyle they are living. Apart from social withdrawal, it has revealed that many hikikomori cases have a sedentary lifestyle, which may harm both their mental and physical health [5,19]. Previous studies have been mainly focused on the psychological and social aspects of hikikomori, but rarely investigated their physical health. A recent local study suggested that a longer social withdrawal period led to a better Quality of Life (QoL) in terms of physical health, psychological health, social relationships and environment, amongst hidden youths recruited from online social networks [20]. Therefore, in the present study, with the focus on physical health and with the aim to establish the first health profile for hikikomori, a cross-sectional study was conducted to explore how well the physical health aspects were of young people who were living in “hikikomori” lifestyle and to measure their lifestyle patterns. Secondarily, health status parameters of such hikikomori cases were compared with those of new onset practitioners.

## 2. Materials and Methods

### 2.1. Target Participants

Hikikomori youths living in Hong Kong (HK) were recruited according to the following inclusion criteria: (1) HK residents of Chinese ethnicity; (2) aged 13–34; (3) not working or attending school; (4) persistent withdrawal for >6 months; and (5) with a social network index (SNI) score of <2. The following individuals were excluded: (1) working in home-based offices; (2) living in an institution or hostel during the past 6 months; (3) part-time students or self-studying; (4) having chronic physical illnesses, severe injury, and/or disability; (5) with psychotic and associated symptoms as screened using the Psychotic Screening Module of the Structured Clinical Interview for Diagnostic and Statistical Manual (DSM) Disorders Axis I (SCID-I); or (6) medically diagnosed with major emotional disorders. However, the criterion of excluding all personal relationships (including friendships) was removed, because in the era of technologies hikikomori can communicate in Internet forums to make online friends, achieve virtual intimacy, and even have close physical friends, all of which are common. Participants who have fulfilled all the above criteria but exhibited persistent withdrawal for <6 months at recruitment were classified as “newly onset cases”; however, before data analysis all cases were confirmed the fulfillment of the 6-month withdrawal criterion as hikikomori cases.

### 2.2. Recruitment and Interview Procedures

Ethical approval (Reference: HSEARS20151126002) was obtained from the Human Subjects Ethics Committee of the HK Polytechnic University. This study was collaborated with the core hidden youth social services in HK, which included nine youth service teams to cover all residential districts as operated by different non-profit organizations. This service network involved 30 social workers which each had to take care of 7–15 clients, hence in total not more than 400 hikikomori cases were currently cared for by the social services, which represented less than 2% of the population-in-need. Such services mainly provide regular visits (once every 1–2 months), social counseling and life planning to encourage the clients to get back to the society. However, social workers would not provide any mental health treatment but refer the cases with psychological needs to relevant medical professionals, and those cases were not recruited in this study. Potential participants were initially invited to participate by their case social workers (already had a trustful relationship). Then the researcher (interviewer) was accompanied by the corresponding social worker to pay a home visit to a potential participant. Following introduction of the researcher by the social worker and the informed consent procedure, the social worker would leave the place temporarily to allow the interview to take place. Psychotic status and eligibility were first assessed through a quick face-to-face interview using the screening questionnaire. Ineligible participants were excluded and the interview was immediately terminated. Eligible participants were then proceeded with the physical measurements and followed by completing a set of self-administered questionnaires. The whole procedure took around 45–60 min to complete. A cash voucher was given to the participants at the end as an incentive. To avoid selection and information bias, the accuracy of the data regarding the eligibility was cross-checked against the case record from the social worker. All researchers were well trained, particularly a 20-h training was provided for the semi-structured SCID-I with the use of the instrument training kit as specified by the developer. Inter-rater reliability was assessed prior to the data collection until satisfactory agreement was achieved among all data collectors.

### 2.3. The Instrument and Measurements

The instrument used in this study adopted a set of established scales. The socio-demographics section assessed: (i) the eligibility of subject in terms of psychiatric status (by SCID-I), age, residential status, working or schooling status, and length of social withdrawal; and (ii) collected information about financial condition, smoking habits, usual daily activities pursued such as surfing the Internet, reading comics, and watching animation. The mental health section adopted: (i) the Chinese 14-item Perceived Stress Scale (PSS-14) for assessing the degree to which hikikomori individuals perceived their lives as stressful; (ii) the Chinese Beck Depression Inventory-II (BDI-II); and (iii) the Chinese State Anxiety Scale of State-Trait Anxiety Inventory (STAI-Y1) for assessing the trait state of anxiety. The lifestyle section mainly evaluated the degrees of distortion on way of living by using: (i) the Chinese Godin Leisure-Time Exercise Questionnaire (GLTEQ) to assess the frequency with which an individual engages in different levels of physical activities; (ii) the Chinese Pittsburgh sleep quality index (PSQI) to measure sleep quality; (iii) “How healthy is your diet? Questionnaire” [21] to measure the number of servings and frequency with which an individual eats certain types of food that the general population normally eats. The social health section assessed both the social and family supports by using: (i) the modified Berkman-Syme Social Network Index (SNI) to measure social connectedness; (ii) the Chinese Interpersonal Support Evaluation List—Short version (ISEL) for assessing appraisal, belonging, tangible dimensions, and the relationship dimension of Chinese Family Environment Scale (CFES) to assess the three key subscales, namely cohesion, expressiveness, and conflict.

The Chinese SF-36 Physical Functioning Subscale (PF-10) was adopted for assessing the physical functioning. Anthropometric measurements including body weight, height, and waist and hip circumference were recorded and used for calculating the Body Mass Index (BMI) and waist-hip ratio. The widest possible head circumference was measured by using a non-stretchable tape. The length and width of ears were measured by using a caliper. The blood pressure (BP; systolic and diastolic values) and pulse rate were measured by using an automatic oscillometric blood pressure monitor (Microlife BP A200 AFIB, Espenstrasse, Switzerland). This device was also equipped with atrial fibrillation (AFIB) detection to suggest the risk of stroke. Blood pressure was measured twice, each 5–10 min apart, and the average value was taken. In case of the two BP readings had a discrepancy over 10%, a well-trained nursing student would use a mercury sphygmomanometer and stethoscope to measure the final BP values. Any positive AFIB indications were repeated the measure when participants had completed the questionnaire administration, and only recorded as positive if both measurements were positive and the participants were recommended to seek for medical help as soon as possible. Respiratory rate was taken by counting the number of breaths for one minute by counting how many times the chest rises.

Internal consistency reliability of the instrument was assessed with 78 youngsters aged 19–23. Cronbach’s alpha vales were 0.80 for PSS-14, 0.88 for BDI-II, 0.91 for STAI-Y1, 0.82 for SF-26 (PF-10), 0.70 for exercise, 0.70 for PSQI, 0.73 for dietary, 0.77 for social connection, 0.74 for social support, and 0.71–0.77 for the three family relationship dimensions.

### 2.4. Data Processing and Analysis

Data collected was analyzed using SPSS Statistics 22.0 (IBM, Armonk, NY, USA). All filled sets of questionnaires were coded. Privacy information that might allow identifying a participant such as name, identity card number, and phone number were not entered into the SPSS data set, but input separately as an excel file encrypted with a password and stored on a separate computer. With regard to demographic data, frequency and percentage were computed for each of the binary or categorical variables (for example gender). Mean and standard deviation (SD) were computed for continuous variables, for example time spent on sleeping. The composite scores were computed for all components scales of the instrument according to the corresponding scoring schemes, and interpreted following the instrument manuals. Means and SD of the composite scores as well as the remaining outcome physical health variables (such as anthropometric and BP measures) were also computed and transformed into the reporting values if necessary. The variables were compared between subgroups of hikikomori cases and newly onset cases by using chi-squared test for those variables with two categories and Students’ *t*-test for those continuous variables. Pearson’s correlational analysis was performed to determine the association between the variables.

## 3. Results

### 3.1. Demographics and Living Lifestyle

From September 2016 to April 2017, a total of 172 hikikomori were initially screened by their case social workers for eligibility, and 104 participants (a success rate of 60.5%) were referred to participate and had completed the set of questionnaires and all anthropometric and physical measurements. Their demographic characteristics were summarized in Table 1 with mean age of 19.02 year-old (SD = 3.62; ranged 13–31) at recruitment and a male-to-female ratio of 3:2. They had been living as hikikomori for 16.14 months (SD = 20.16; ranged 3–72 months), and were divided half-and-half into the newly onset cases (3–6 months) and hikikomori cases (>6 months) groups. Prior to the data analysis, all newly onset cases were confirmed in follow-up for persistent withdrawal beyond 6 months. A vast majority of the cases (96%) was dependent on their family for housing and living. Older cases indicated to be more hesitant to communicate with strangers (*p* < 0.05), while they were more dependent on family’s financial support (*p* < 0.05). Two cases indicated they were neither living alone nor with family, including one older case who reported living with relatives other than the immediate family, and a newly onset case reported living in a close friend’s home. Regarding the financial support, 32.7% of the newly onset cases and 11.5% of the older cases indicated that they did not ask for money from anyone, including their family. Although the questionnaire did not have particular items to address the reason for subjects who did not require financial support from others, the researchers verbally asked and documented on the kind of self-financial support they acquired. Multiple explanations were captured from some cases who have reported: living with money left over from a job they had recently quit; supported themselves with their own savings instead of asking family for money; or did not need to spend money because they stayed at home all day. None of them were working remotely, because it was set as an exclusion criterion. However, no information could be provided on whether any of the cases were being supported by social welfare.

Lifestyle patterns of the two subgroups are compared in Table 2. Overall, about half of the participants (45.2%) were living with a sedentary lifestyle in accordance with their Gobin weekly leisure activity scores, which was significantly (*p* < 0.01) more common in the older cases. Participants performed light exercise three times and moderate exercise 1.5 times in an average week, but rarely performed exercise at a strenuous level (Table 2). Participants slept almost 8 h per day and spent most of their awake time staying at home and using electronic devices. They spent 1–3 h on eating but the diets were relatively unhealthy with a “How healthy is your diet” score of 12.6 (SD = 4.85) out of 33 (Table 2).

In an average week, 91% of participants had consumed fast food at least once and 77% consumed different kinds of sweets. Over half consumed sugary drinks every day, and particularly soda and caffeinated drinks were the most mentioned drinks consumed. Furthermore, 80% of the participants consumed one or less of vegetables and fruits serving per day while 83% consumed four types or less per month. Results also indicated that the majority (74%) slept poorly, with a mean Global PSQI score of 6.85 (SD = 3.36). Lowest scores were rated in subjective sleep quality, sleep latency, and sleep disturbance. Furthermore, hikikomori youths of this study demonstrated a certain degree of asocial behavior as measured by SNI and ISEL, and typical with perceived stress and state anxiety at moderate levels and borderline clinical depression, but no significant differences were observed between the older and newly onset cases in all psychosocial health measurements (data to be published elsewhere).

### 3.2. The Physical Health Profile

A vast majority of participants were indicated to be at good physical functioning with a SF-36 subscale score >80 (Table 3), but a significant proportion exhibited problems with body weight and blood pressure.

Up to 70% of the participants exhibited the division of their body weight into two opposing extremes as either underweight or overweight/obese. Underweight was dominated among the newly onset cases, specifically 46% was rated as “underweight” according to the BMI classification and 39% rated “below standard” according to the body fat classification (Table 3). In contrast, the older cases were significantly heavier (*p* < 0.001) in weight and higher in other parameters including BMI (*p* < 0.01), waist circumference (*p* < 0.001) and waist-to-hip ratio (*p* < 0.01). Overall, 38% of the older cases were classified as obese in accordance with body fat percentages while the majority were at the mildly obese level. Consistently, 48% of older cases were rated overweight or obese according to the BMI criteria, which was further broken down into 7.7% overweight, 21.2% pre-obese and 19.2% obese. No significant difference was observed between the two groups in other anthropometric variables (Table 3).

According to the JNC7 classification, 16 (15.4%) and 33 (31.7%) participants were found to have hypertension and prehypertension, respectively, with mean SBP/DBP of 141.31/90.69 (SD = 10.29/5.40) mmHg and 125.85/79.09 (SD = 8.92/5.28) mmHg. Significantly higher systolic and diastolic values were shown in the older cases, whereas the incidence of hypertension and prehypertension were 3-fold and 1.5-fold of the newly onset cases, respectively. Half of the hypertensive cases were the isolated diastolic type while 31% were the isolated systolic type (80% older cases) and the 19% were systolic-diastolic type (all older cases). Only one stage 2 hypertensive case was identified who had been living as hikikomori for 24 months. Age was the only demographic characteristic that correlated with the SBP (*r* = 0.27; *p* < 0.01) and DBP (*r* = 0.34; *p* < 0.01) levels. However, positive and significant correlations (*r* = 0.28–0.63; *p* < 0.01) were observed between the BP levels of participants and all obesity index parameters measured (Table 3). None of the hypertensive cases identified in this study was found to have a positive AFIB.

## 4. Discussion

To our best knowledge, this is the first study conducted to measure the physical health of hikikomori along with their living lifestyle. Hong Kong youths living with a hikikomori lifestyle were found to have a high incidence of hypertension and prehypertension, which were believed to be correlated with the weight gains during the course of their hermitic behavior. Results suggested that the length of hikikomori duration was associated with a shift of body weight from underweight to overweight and obesity, which has also signified the problem of elevated blood pressure. Such physical manifestations seemed to be at least partially associated with the sedentary lifestyle that was commonly shared among the hikikomori cases, in addition to their unhealthy dietary habits and distorted sleep patterns.

Participants of this study shared similar asocial behavior with a few previous studies conducted in other places [1,4,5], which were also co-morbid with negative emotional states more commonly with depression, anxiety and distress. This study observed an overall high prevalence of 15.4% for hypertension. This was slightly higher than the 12.6% local prevalence including all diagnosed hypertensive cases in adults as reported by the Census and Statistics Department of HK in 2014 [22], but much lower than the adjusted prevalence of 32% after including hidden hypertensive cases within the community as reported in a recent local large-scale cohort study [23] In fact, according to a large pooled analysis involving 19.1 million adults in the past two decades, decreasing trends were observed in most developed countries when hypertension was standardized by age, which concluded the global increase of hypertensive number is a net effect of population growth and ageing [24]. In contrast, the trends were commonly demonstrated to be increasing in different populations of adolescents and young adults since 1960s [25,26,27,28]. In HK, children and adolescents at age 9–15 years had recorded the range of 12.0–13.8% on hypertensive prevalence [29]. Whilst Taiwanese at age 20–29 years were reported to have a prevalence of 15.1% for men and 3.7% for women [30]. Despite the prevalence of hypertension in hikikomori was found to be roughly equivalent to that of their age-matched groups, more concerns were caused by another one-third (31.7%) of hikikomori cases that were identified as “prehypertension”, where the prevalence for the newly onset and older cases were found as 25.0% and 38.5%, respectively. These prevalence rates were not much below the 42.7–50.6% prevalence reported amongst the adults at age 20–50 [30,31,32], but much higher than the 12–17% prevalence reported in children and adolescents [33]. The hikikomori cases of this study consisted of a mixture of adolescents and young adults at 4:6 ratio with age ranged 13–34 years, the prevalence should be somewhere in-between the reported prevalence of adolescents and young adults. Although pre-hypertension is rarely investigated amongst the younger populations and no local study was available for comparison, the high prevalence of approaching 40% in the older hikikomori cases should be self-judgmental as a sub-health group. The risks of transiting prehypertension into hypertension and other cardiovascular complications and metabolic disorders have been well documented [31,34,35,36]. Blood pressure has demonstrated as an important indcator of the cardiovascular system in adolescents and young adults, where a number of cardiometabolic parameters could be significantly altered at the prehypertensive stage, and hence supporting the notion of pharmacologic interventions earlier [37]. Looking more-in-depth into the hypertensive types, hypertension occurring at younger ages are more commonly belonging to the isolated diastolic type, because an increase of systolic BP is often caused by changes of arterial stiffness that should be more frequently happened with aging but unexpected at younger ages [34,38]. Besides being a primary target for antihypertensive therapies, systolic BP also carries predictive value for cardiovascular risk [39]. Systolic hypertension was known to have higher odds for cardiovascular diseases and stroke [40]. Despite no positive AFIB was discovered in any of the hypertensive cases in this study which excluding quivering or irregular heartbeat to suggest no immediate risk of stroke [41], the high prevalence of prehypertension and involvement of systolic BP elevation in half of the cases caused much worry.

Furthermore, BP levels of hikikomori cases herein were also found to be associated with various adiposity measures, whereas many participants were engaged in sedentary lifestyle that characterized by insufficient physical activities and snacking on unhealthy food items. Many studies have demonstrated the positive correlation between hypertension and obesity [25,27]. In younger populations, BMI and waist circumference have been accepted as two independent risk factors predictive for the occurrence of hypertension [42,43,44]. The Wellness Population of Youth Study [29] recently reported when compared with worldwide data, children and adolescents of Hong Kong had more severe body weight problem with 5.4–15.1% obesity and 20.8–25.9% overweight. Hikikomori cases of this study were also shown to have bodyweight problems, particularly with overweight/obesity in approximately 20.0% of the newly onset cases but almost half of the older cases. Concurrently, both hypertension and prehypertension were significantly more prevalent in older cases who have been engaged longer as hikikomori. These observations implied the poor physical health conditions with high prevalence of weight gain and raised BP were at least partially related to the hikikomori’s living lifestyle. Nonetheless, the daily activities of hikikomori cases identified herein were consistent with the previous reported top solitary activities pursued by young socially withdrawn people such as surfing the Internet, chatting on-line with strangers, and sitting in a corner, all of which were sedentary in nature [5,45,46]. Sedentary lifestyle itself is already a known risk factor for hypertension and other cardiovascular complications [47,48,49,50], whereas sedentary working pattern was even proposed as a strong predictive factor for developing into hypertension among individuals who were pre-hypertensive [31]. Adults in sedentary occupations were shown to have higher odds of cardiovascular diseases and diabetes [51]. Beside occupational, sedentary behaviors for leisure such as frequent television viewing have also been associated with an adverse profile of raised BP, BMI, and several cardiovascular and diabetes biomarkers [52]. Hikikomori might not be more sedentary than the average individual who works at a computer or watches television all day, but no study has been conducted comparing the hikikomori with other occupational or non-occupational groups. However, in addition to sedentary behavior, hikikomori have several other lifestyle factors that may also contribute to hypertension and obesity. Participants of this study consumed sweet snacks and sugary drinks frequently were also highly engaged in fast food diets, often high in fat, low in fiber and high in sodium. Proper nutritional intake during young adulthood is known to be important for preventing excessive weight gain, in order to maintain physical health and reduce the risk for future disease development. Many young people attempted poor eating habits because they have less knowledge to link lifestyle choices with obesity or the consequence to future morbidities [53]. Young adults have less food preparation skills while consumption of fast food is common [54]. Frequent use of fast food causes accumulation of fat and cholesterols, especially triglycerides that could lead to narrowing of blood vessels and atherosclerosis that may also contribute to elevated BP [55,56]. High salt intake is not only a proven cause of BP elevation, but it is also associated with obesity independent of energy intake [57]. Excessive energy intake and insufficient physical activities could be a major cause of weight gains by time, which eventually leads to obesity [57,58]. Furthermore, the poor sleeping quality of current participants was consistent with previous report that hikikomori tended to sleep at extremely late night hours or during the day [59]. Very frequent and prolonged use of computers and electronic devices at home as the top activities amongst the hikikomori cases could be associated with poor sleep quality, which coincided with the strong association between sleep quality and daytime function with the use of technologies [60,61]. Irregular sleep-wake patterns can interfere with the light–dark cycle and the circadian clock in the human body, which could have deleterious effects on different aspects of health and quality of life [62]. Clinical significance of human circadian rhythms was reviewed [63], which has highlighted the negative impacts of disrupted sleeping cycle on cardiovascular regulation associated with BP levels. Another study discussed how irregular sleep-wake rhythm of hikikomori could be associated with physical problems such as headaches, neck, back, or muscle pain, and gastrointestinal problems [59]. However, such physical parameters have not been measured in the current study, and are deemed to be further investigated. Putting altogether, the physical traits of hikikomori with raised BP and obesity were likely to be related to their distorted lifestyle. The current study suggested longer the social withdrawal of the hikikomori cases, more distorted on lifestyle behavior and poorer the physical health conditions with weight gain and raised BP. This notion seemed to be contradictory with the report of Chan and Lo [20], which was relied on the WHOQOL-BREF questionnaire that the longer the length of social withdrawal was indicated to be associated with a better quality of life including physical health. The authors explained prolonging the social withdrawal would allow more time to those hidden youths to solve their problems by receiving support and recognition from their hidden youth peers through the Internet with better the family relationships, but an opposite outcome was revealed if such social connections failed to be established during the social withdrawal [20]. With the focus on the physical domains measured in those hidden youths, the authors reported only the domain scores of the instrument used and correlations with the length and level of social withdrawal, but did not discuss in details about the measured facets that in what entities the better physical health were observed, such as pain and discomfort [20].

This study has several strengths. First, this is the first of its kind to explore this hidden population by including empirical physical measurements. The physical assessments were not only beneficial in objective measurements to strengthen the evidence, but it was also found to be important to arise the interest and awareness of participants to concern more of their health or at least to adopt a less “hikikomori-type” lifestyle. Social workers also found such kind of physical measurements could help to engage their clients better and create more dialog with their clients. Second, samples were recruited from multiple centers that were operated by different social service teams. This approach covers well HK’s residential areas, which allows a representative sampling and reducing selective and geographical bias. Third, during the home visits, interviewers were first introduced by the case social workers who have already established a trustful relationship with the participants. This procedure was found to be effective in enhancing the successful rate of subject recruitment, which is particularly important for those were originally asocial. It avoided miscommunications and misunderstanding, which resulted in a very quick buildup of bonding between the participant and interview to facilitate the interview process and physical assessment. Fourth, all physical assessments were conducted by well-trained registered nurses or nursing students who have sufficient knowledge to ensure accurate measurements, and at a more appropriate position to handle health needs or enquiries that may be brought up by the participants. 

There are also several limitations in this study, owning to the hidden nature of the target participants who are basically unreachable, so subject recruitment is considered as the most difficult part of the study. This led to a small sample size as a major limitation. However, the sample size was sufficient to be divided evenly into two subgroups, which was important to achieve statistical significance when certain measured variables were compared. Furthermore, although participants of this study were recruited from multiple centers, sampling through a single agent i.e., social work is also considered as a major limitation because many hidden cases still could not be reached. It is suggested that other agencies such as secondary schools, student residency of universities, other family-based services, medical units, and even relevant online forums and social media platforms can also be approached for sampling in the future studies. In relation to the recruitment issue with a single agent, it is also a limitation that the subjects of this study were all engaged with social work service, and as mentioned above the population represented less than 2% of all hikikomori cases of Hong Kong. However, these samples were the only accessible, available and allowable for physical health measurements and face-to-face measurement, at least before collaborations with other agencies can be established. Furthermore, it is also important to be clarified that social workers may intervene the samples in different ways but such interventions should not affect directly the outcomes measured herein, and more importantly social service of Hong Kong do not include mental health treatments but referral will be made if any medical help is needed and those with mental health issues were excluded in this study.

## 5. Conclusions

A significant portion of Hong Kong’s hikikomori youths is living in adverse physical health conditions, particularly raised blood pressure and weight gains. Although the design of this cross-sectional study was inappropriate for examining the cause-and-effect relationship between such health conditions and the lifestyle the hikikomori were living, both the blood pressure and bodyweight problems were demonstrated to be more serious in the cases that have lived longer as hikikomori. The hikikomori lifestyle is typically characterized by sedentary behavior, poor eating habits, and distorted sleeping quality that were all well proven factors contributing to both hypertension and obesity. Overall, the hikikomori lifestyle should be considered a risk behavior that may harm the younger generation physically by promoting obesity and increasing the chance of hypertension and possibly other chronic illnesses. This research may also have a benefit to inform the caregivers of hikikomori such as parents and social workers about their potential health risks, and this may also alert the hikikomori youths to become more collaborative and worry about their own health. Currently, a longitudinal study is undergoing to follow-up on this high-risk group on measuring the changes in various health aspects.

## Figures and Tables

**Table 1:** Demographic characteristics of hikikomori youths living in Hong Kong.

Variables		Total (*N* = 104)	Newly Onset Cases (*N* = 52)	Older Cases (*N* = 52)	χ^2^
					*p*-Value
		Number (Percentage)			
Gender	Male	62 (59.6)	29 (55.8)	33 (63.5)	0.424
	Female	42 (40.4)	23 (44.2)	19 (36.5)	
Age (years)	13–17	41 (39.4)	24 (46.2)	17 (32.7)	0.288
	18–24	54 (51.9)	25 (48.1)	29 (55.8)	
	25–34	9 (8.7)	3 (5.7)	6 (11.5)	
Mean ± SD		19.02 ± 3.62	18.42 ± 3.59	19.62 ± 3.5	0.127
Duration of being hikikomori (Mths), mean ± SD		16.14 ± 20.16	3.63 ± 1.09	28.65 ± 22.36	<0.001
Living	Alone	2 (1.9)	0 (0)	2 (3.8)	0.257
	With immediate family ^	100 (96.2)	51 (98.1)	49 (94.2)	
	With relatives ^/friends	2 (1.9)	1 (1.9)	1 (1.9)	
Residential	Self-owned/self-rented	2 (1.9)	1 (1.9)	1 (1.9)	0.382
	Not self-owned/Not-self-rented	102 (98.1)	51 (98.1)	51 (98.1)	
Financial source	Self	23 (22.1)	17 (32.7)	6 (11.5)	0.024
	Family/relatives	80 (76.9)	34 (65.4)	46 (88.5)	
	Refused to answer	1 (1)	1 (1.9)	0 (0)	
Self-perceived hesitation on communication with strangers ^#^		30 (28.8)	10 (19.2)	20 (38.5)	0.030

Note: ^#^ Self-perceived hesitation including direct or face-to-face communication with strangers; ^ Immediate family members include parents, spouse, brothers, sisters, sons and daughters; Extended family members other than those listed above are referred as relatives.

**Table 2:** Lifestyle living with the hikikomori of Hong Kong.

Variables		Total (*N* = 104)	Newly Onset Cases (*N* = 52)	Older Cases (*N* = 52)	*t*-Test *p*-Value
		Mean ± SD			
**No. of hours staying at home**		19.11 ± 4.40	18.19 ± 4.83	20.02 ± 3.76	0.425
**Daily activities at home (hours)**					
Sleeping		7.83 ± 1.99	7.54 ± 1.89	8.12 ± 2.06	0.140
Using Computer		5.09 ± 4.97	4.44 ± 4.37	5.73 ± 5.48	0.188
Using mobile phone/tablet		3.11 ± 5.03	3.10 ± 5.11	3.12 ± 5.00	0.985
Eating		1.90 ± 1.03	1.77 ± 0.942	2.04 ± 1.10	0.183
Reading comics/animation		0.95 ± 2.38	1.00 ± 2.94	0.90 ± 1.68	0.838
Watching TV		0.90 ± 1.13	0.73 ± 1.07	1.08 ± 1.17	0.118
Other readings		0.80 ± 1.44	0.83 ± 1.45	0.77 ± 1.44	0.839
Other activities (not disclosing)		0.65 ± 2.09	0.35 ± 0.86	0.96 ± 2.81	0.135
Idling/facing the wall		0.40 ± 0.99	0.38 ± 0.72	0.42 ± 1.21	0.844
**Sleeping quality**					
Global PSQI score		6.85 ± 3.36	6.94 ± 3.27	6.75 ± 3.48	0.772
Poor sleeper (PSQI >5), *N* (%)		77 (74.0)	41 (78.9)	36 (69.2)	0.263 ^#^
**How healthy is your diet? (Max. score = 33)**		12.57 ± 4.85	12.73 ± 4.51	12.40 ± 5.20	0.733
**Physical activity**					
Weekly leisure activity score		24.87 ± 31.81	29.69 ± 35.58	20.06 ± 27.02	0.123
Level, *N* (%)	Active	38 (36.5)	23 (44.2)	15 (28.9)	0.002 ^#^
	Moderately active	19 (18.3)	14 (26.9)	5 (9.6)	
	Insufficiently active/Sedentary	47 (45.2)	15 (28.9)	32 (61.5)	
**Smoking, *N* (%)**	Never	81 (77.9)	39 (75)	42 (80.8)	0.669 ^#^
	Quitted	10 (9.6)	5 (9.6)	5 (9.6)	
	Current smoker	13 (12.5)	8 (15.4)	5 (9.6)	

Note: ^#^ Determined by using chi-squared test.

**Table 3:** A summary of anthropometric, physical functioning and physical health in hikikomori.

Variables	Overall (*N* = 104)	Newly Onset Cases (*N* = 52)	Older Cases (*N* = 52)	*t*-Test *p*-Values
	Mean ± SD			
**Anthropometrics**				
Height (cm)	164.0 ± 9.9	162.4 ± 10.4	165.5 ± 9.2	0.101
Weight (Kg)	60.6 ± 19.8	53.9 ± 13.9	67.4 ± 22.6	**<0.001**
BMI (Kg/m^2^)	22.3 ± 6.9	20.1 ± 4.7	24.4 ± 8.0	**0.001**
BMI classification, *N* (%)				
Underweight	39 (37.5)	24 (46.2)	15 (28.8)	**0.008 ^#^**
Normal	30 (28.8)	18 (34.6)	12 (23.1)	
Overweight/Obese	35 (33.7)	10 (19.2)	25 (48.1)	
Body fat (%)	21.1 ± 9.3	18.8 ± 8.3	23.3 ± 9.8	**0.014**
Body fat classification, *N* (%)				
Below standard	32 (30.8)	20 (38.5)	12 (23.1)	**0.002 ^#^**
Standard	47 (45.2)	27 (51.9)	20 (38.5)	
Obese	25 (24.0)	5 (18.3)	20 (38.4)	
Waist circumference (cm)	77.6 ± 16.9	71.1 ± 11.9	84.2 ± 18.7	**<0.001**
Non-ideal waist circumference, *N* (%)	28 (26.9)	5 (9.6)	23 (44.2)	**<0.001 ^#^**
Waist-to-hip ratio	0.82 ± 0.09	0.80 ± 0.74	0.85 ± 0.10	**0.004**
**Physical functioning (SF-36)**	83.99 ± 19.01	86.54 ± 17.70	81.44 ± 20.08	0.173
**Physical health measurements**				
SBP (mmHg)	118.0 ± 15.6	113.7 ± 13.1	122.4 ± 16.8	**0.004**
DBP (mmHg)	75.0 ± 10.3	71.7 ± 9.7	78.4 ± 9.8	**0.001**
Pulse pressure (mmHg)	42.8 ± 10.0	42.0 ± 9.0	43.6 ± 10.9	0.435
Pulse rate (per minute)	84.9 ± 14.6	82.5 ± 11.9	87.3 ± 16.6	0.096
Respiratory rate (per minute)	17.7 ± 1.2	17.5 ± 1.1	18.0 ± 1.3	0.060
AFIB, *N* (%)	0	0	0	-
BP classification, *N* (%)				
Normal	54 (51.9)	35 (67.3)	20 (38.5)	**0.020 ^#^**
Prehypertension	33 (31.7)	13 (25.0)	20 (38.5)	
hypertension	16 (15.4)	4 (7.7)	12 (23.0)	

Note: ^#^ Determined by using chi-squared test.
